# Systems for grading the quality of evidence and the strength of recommendations II: Pilot study of a new system

**DOI:** 10.1186/1472-6963-5-25

**Published:** 2005-03-23

**Authors:** David Atkins, Peter A Briss, Martin Eccles, Signe Flottorp, Gordon H Guyatt, Robin T Harbour, Suzanne Hill, Roman Jaeschke, Alessandro Liberati, Nicola Magrini, James Mason, Dianne O'Connell, Andrew D Oxman, Bob Phillips, Holger Schünemann, Tessa Tan-Torres Edejer, Gunn E Vist, John W Williams, The GRADE Working Group

**Affiliations:** 1 Center for Practice and Technology Assessment, Agency for Healthcare Research and Quality, 540 Gaither Rd. Rokville, MD 20852, USA; 2 Community Guide Branch, Centers for Disease Control and Prevention, MS K73, 4770 Buford Highway, Atlanta, GA 30341, USA; 3 Centre for Health Services Research, University of Newcastle upon Tyne, 21 Claremont Place, Newcastle upon Tyne NE2 4AA, UK; 4 Informed Choice Research Department, Norwegian Health Services Research Centre, Pb. 7004 St. Olavs Plass, 0130 Oslo, Norway; 5 Departments of Clinical Epidemiology and Biostatistics and Medicine, McMaster University, 1200 Main Street West, Hamilton, Ontario L8N 3Z5, Canada; 6 Scottish Intercollegiate Guidelines Network, 9 Queen Street, Edinburgh EH2 1JQ, UK; 7 Department of Clinical Pharmacology, Faculty of Medicine and Health Sciences, University of Newcastle, Level 5, New Med 2 Building, Newcastle Mater Hospital, Waratah, NSW 2298, Australia; 8 Department of Medicine, McMaster University, 1200 Main Street West, Hamilton, Ontario L8N 3Z5, Canada; 9 Department of Oncology and Hematology, Università di Modena e Reggio Emilia, Azienda Ospedaliera Policlinico, Via dal Pozzo 41, 41100 Modena, Italia and Centro per la Valutazione della Efficacia della Assistenza Sanitaria (CeVEAS), Modena, Italy; 10 Centro per la Valutazione della Efficacia della Assistenza Sanitaria (CeVEAS), NHS Centre for the Evaluation of the Effectiveness of Health Care, Viale Muratori 201, Modena 41100, Italy; 11 Cancer Epidemiology Research Unit, Cancer Research and Registers Division, The Cancer Council NSW, PO Box 572, Kings Cross NSW 1340, Australia; 12 Centre for Evidence-based Medicine, University Department of Psychiatry, Warneford Hospital, Oxford OX3 7JX, UK; 13 Departments of Medicine and Social & Preventive Medicine, University at Buffalo, State University of New York, ECMC-CC142, 462 Grider St, Buffalo, NY 14215, USA; 14 Global Programme on Evidence for Health Policy, World Health Organisation, CH-1211 Geneva 27, Switzerland; 15 The Center for Health Services Research in Primary Care, HSR&D, Department of Veterans Affairs Medical Center and Duke University Medical Center, 508 Fulton St., Durham, NC 27705, USA

## Abstract

**Background:**

Systems that are used by different organisations to grade the quality of evidence and the strength of recommendations vary. They have different strengths and weaknesses. The GRADE Working Group has developed an approach that addresses key shortcomings in these systems. The aim of this study was to pilot test and further develop the GRADE approach to grading evidence and recommendations.

**Methods:**

A GRADE evidence profile consists of two tables: a quality assessment and a summary of findings. Twelve evidence profiles were used in this pilot study. Each evidence profile was made based on information available in a systematic review. Seventeen people were given instructions and independently graded the level of evidence and strength of recommendation for each of the 12 evidence profiles. For each example judgements were collected, summarised and discussed in the group with the aim of improving the proposed grading system. Kappas were calculated as a measure of chance-corrected agreement for the quality of evidence for each outcome for each of the twelve evidence profiles. The seventeen judges were also asked about the ease of understanding and the sensibility of the approach. All of the judgements were recorded and disagreements discussed.

**Results:**

There was a varied amount of agreement on the quality of evidence for the outcomes relating to each of the twelve questions (kappa coefficients for agreement beyond chance ranged from 0 to 0.82). However, there was fair agreement about the relative importance of each outcome. There was poor agreement about the balance of benefits and harms and recommendations. Most of the disagreements were easily resolved through discussion. In general we found the GRADE approach to be clear, understandable and sensible. Some modifications were made in the approach and it was agreed that more information was needed in the evidence profiles.

**Conclusion:**

Judgements about evidence and recommendations are complex. Some subjectivity, especially regarding recommendations, is unavoidable. We believe our system for guiding these complex judgements appropriately balances the need for simplicity with the need for full and transparent consideration of all important issues.

## Background

Reviewers and users of reviews draw conclusions about the overall quality of the evidence that is reviewed. Similarly, people making recommendations and users of those recommendations draw conclusions about the strength of the recommendations that are made. Systematic approaches to doing this can help protect against errors by both doers and users, and can facilitate critical appraisal and communication of the conclusions that are made.

The GRADE Working Group began as an informal collaboration of people with an interest in addressing shortcomings in systems for grading evidence and recommendations. We report elsewhere a critical appraisal of six prominent systems for grading evidence and recommendations [1]. Based on this critical appraisal and a series of discussions, we reached agreement on the key attributes of a system that would address the major shortcomings that we identified. Based on the critical assessment of existing approaches, the agreement we had reached about the key elements that should be included in an approach for grading the level of evidence and strength of recommendations and our previous experiences we put together a suggestion for a grading system. We then applied the suggested system to a series of examples and discussed and revised the system based on this experience and the consideration of other examples. Examples were selected to challenge our thinking. All of the examples used in this pilot study were questions about interventions. We describe here the pilot study of this system.

The aims of the pilot study were to test whether the approach is sensible relative to diverse examples of evidence and recommendations, and to agree on necessary changes to the approach, decision rules, and changes in how the evidence profiles used in the pilot study were constructed. The revised approach is described elsewhere [16].

## Methods

Seventeen people independently judged the quality of evidence, the balance between benefits and harms, and the formulation of a recommendation for 12 examples. The 17 judges all had experience using other approaches to grade evidence and recommendations.

### Evidence profiles

For each example we prepared an evidence profile. Each evidence profile was made based on information available in a systematic review and consists of two tables, one for quality assessment of the available information and one table that presents a summary of the findings (Table 1 and Table 2). For the purpose of testing our grading approach in this pilot study we made the assumption that the systematic reviews that we used were all well conducted. The examples we used and presented here were selected to test our new approach, not with an intention of making actual recommendations for a specific setting based on up-to-date systematic reviews. The quality assessment table was designed such that the quality of each outcome was evaluated separately. For each outcome, the table contained information regarding the number of studies that had reported the outcome, information about the study design (RCTs or observational studies) and the quality of the studies that reported on that outcome (was there any limitations in the design or conduct of these studies). Also included in the quality assessment table was information about the consistency of the results across studies for each outcome and information regarding directness of the study population, outcome measure, intervention and comparison. The summary of findings table was also designed such that each outcome was presented separately. For each outcome information are presented about both the experimental and the control group patients, for dichotomous outcomes the number of events and the total number of participants, and for continuous outcomes means (standard deviation) and the number of patients were presented. Also included in the summary of findings table is information about the effect, relative effect (95% confidence interval) and absolute effect for each outcome.

**Table 1:** Example of an evidence profile quality assessment given to the evaluators for them to grade in the pilot study. Example question: Should depressed patients in primary care be treated with SSRIs rather than tricyclics?

Outcome: **Depression severity **(measured with Hamilton Depression Rating Scale)				
Studies	Design	Quality	Consistency	Directness

8 trials Citalopram 38 trials Fluoxetine 25 trials Fluvoxamine 2 trials Nefazodone 18 trials Paroxetine 4 trials Sertaline 4 trials Velafaxine	RCTs	No serious flaws	No important inconsistency	Some uncertainty about relevance (outcome measure)

Outcome: **Transient side effects **(drop-out from 6 week treatment)				

8 trials Citalopram 50 trials Fluoxetine 27 trials Fluvoxamine 4 trials Nefazodone 23 trials Paroxetine 6 trials Sertaline 5 trials Velafaxine	RCTs	No serious flaws	No important inconsistency	Some uncertainty about relevance (outcome measure)

Outcome: **Poisoning fatalities**				

Office for National Statistics (British)	Observational data (national statistics)	Serious flaw, population based Reporting bias	Only one study	Direct

**Table 2:** Example of an evidence profile summary of findings given to the evaluators for them to grade in the pilot study. Example question: Should depressed patients in primary care be treated with SSRIs rather than tricyclics?

**Outcome**	SSRI	tricyclics	Effect		Quality	Relative importance
					
			Relative (95% CI)	NNT/NNH		
Depression Severity	5044 patients	4510 patients	WMD 0.034 (-0.007 to 0.075)	No difference		
Transient side effects	1948/7032 (28%)	2072/6334 (33%)	RRR 13% (5% to 20%)	20		
Poisoning fatalities*	1/100,000 per year of treatment	58/100,000 per year of treatment	RRR 98%	1754		

* Uncertainty about baseline risk: Fatality data may be influenced by which pills are given to whom, and it is uncertain if changing antidepressant would deter suicide attempts

Instructions and a form for recording each judgement were included with each example [see Additional file 1]. The judges were instructed to apply the approach without second guessing the information presented in the evidence profile or the approach. They were asked to note problems that they encountered and judgements that did not make sense to them when they adhered to the approach as instructed.

### Questions and judgements

The 12 examples were selected to include a variety of health care interventions, types of evidence and types of recommendations. The questions that were posed in the 12 examples were:

• Should depressed patients in primary care be treated with SSRIs or tricyclics? [2]

• Should patients with atrial fibrillation be treated with warfarin or aspirin for prevention of stroke? [3]

• Should patients with pain believed to be due to degenerative arthritis be treated with non-steroidal anti-inflammatory drugs (NSAIDs) or paracetamol? [4]

• Should patients who have had a myocardial infarction be given antiplatelet therapy to reduce all cause mortality? [5]

• Should patients who have had a myocardial infarction be offered exercise rehabilitation? [5]

• Should patients with deep venous thrombosis be treated with Low Molecular Weight Heparin (LMWH) or IV unfractionated heparin for prevention of pulmonary embolism? [6]

• Should antibiotics be used to treat acute maxillary sinusitis? [7]

• Should BCG vaccine be used to prevent tuberculosis? [8]

• Should surgical discectomy be recommended for patients with sciatica due to lumbar disc prolapse? [9]

• Should community water fluoridation be used to reduce dental caries? [10,11]

• Should distribution of child safety seats and education programs be used to increase correct use of child safety seats? [12]

• Should hormone replacement therapy be given to prevent cardiovascular heart disease in healthy post menopausal women? [13]

For each example each person made judgements about;

• the quality of evidence for each outcome, scored as high, intermediate, low, or very low;

• the relative importance of each outcome, scored as critical to the decision (7–9), important but not critical to the decision (4–6), or not important to the decision (1–3);

• the overall quality of all the critical outcomes, scored as high, intermediate, low, or very low;

• the balance between benefits and harms, scored as net benefit, trade offs, uncertain net benefit, or not net benefit; and

• the recommendation, scored as do it, probably do it, toss up, probably don't do it, or don't do it.

For each example the judgements made by all 17 people were collected and summarised as illustrated in Table 3. Disagreements were discussed at a meeting attended by 15 of the 17 judges. Because of a lack of time, the last two examples were discussed at another meeting attended by six of the 17 judges, but all 17 raters provided judgements for all of the 12 examples. For each example the kappa agreement was calculated [14] for the 17 graders across the four levels for the quality of evidence across outcomes for each example (number of outcomes per example range from two to seven), across all outcomes (46) and for the judgements about overall quality of the evidence (12).

**Table 3:** Summary of the judgements made by the 17 evaluators for Example 1 of the pilot study. Should depressed patients in primary care be treated with SSRIs rather than tricyclics?

Rater	Quality of outcome			Relative importance of outcome			Overall quality	Balance benefits vs harm	Recommendation
	Depression severity	Transient side effects	Poisining fatalities	Depression severity	Transient side effects	Poisining fatalities			

1	H	H	Vl	7	9	9	H or Vl	Uncertain net benefit	Don't do it
2	M	M	Vl	9	6	7	Vl	Net benefit	Probably do it
3	H	H	M	8	7	9	H	Uncertain net benefit	Toss up
4	H	H	L	6	5	6			
5	M	M	M	9	6	8	M	Net benefit	Do it
6	M	M	Vl	9	6	9	Vl	Net benefit	Do it
7	M	M	L	8	7	8	L	Net benefit	Do it
8	H	H	Vl	9	5	3	H	Net benefit	Probably do it
9	M	M	L	9	6	8	L	Net benefit	Probably do it
10	M	M	L	9	7	8	L	Net benefit	Probably do it
11	H	H	L	8	5	7	L	Trade offs	Probably do it
12	H	H	L	8	5	7	L	Trade offs	Probably do it
13	M	H	M	9	7	9	M	Net benefit	Do it
14	M	M	L	9	9	5 OR 9	M	Net benefit	Probably do it
15	M	M	Vl	9	6	8	Vl	Uncertain net benefit	Toss up
16	M	M	Vl	9	5	9	Vl	Not net benefit	Don't do it
17	M	M	M	9	9	9	M	Net benefit	Toss up

### Sensibility and understandability

After grading all 12 examples, the judges were asked 16 questions regarding the sensibility and understandability of the approach. Each question consisted of a statement and five response options: strongly disagree, disagree, not sure, agree, and strongly agree. Eleven people completed this questionnaire. The questionnaire was adapted from Feinstein [15] and the 16 statements were:

1. The approach is applicable to different types of interventions, including drugs, surgery, counselling, and community-based interventions.

2. The approach is clear and simple to apply

3. The information that is needed is generally available.

4. Subjective decisions are generally not needed.

5. All of the components included in each of the five types of judgements should be included

6. There are not important components that are missing for any of the five types of judgements.

7. The ways in which the components are aggregated for each of the five types of judgements are clear and simple.

8. The ways in which the included components are aggregated are appropriate for each of the five types of judgements.

9. The categories are sufficient to discriminate between different grades for each of the five types of judgements.

10. The approach successfully discriminates between different grades of evidence.

11. The approach successfully discriminates between different grades of recommendations.

12. The overall quality of evidence is clear and understandable.

13. The balance between the benefits and harms is clear and understandable.

14. The recommendation is clear and understandable.

15. The way in which the overall quality of evidence was graded is better than other ways of doing this with which I am familiar.

16. The way in which the recommendation was graded is better than other ways of doing this with which I am familiar.

## Results

### Quality of evidence for each outcome

The quality of evidence for each outcome as assessed by the 17 graders are shown in Table 4. Much of the disagreement was due to lacking information in the evidence summaries that we prepared based on the information available in the chosen examples. We agreed that the evidence summaries should include footnotes explaining the basis for judgements about study quality, consistency and directness. We also agreed that it was necessary to include information about baseline risk and the setting as part of the background information since different assumptions about these factors also explained some of the disagreement. It was possible to reach a consensus about the quality of evidence for most outcomes when we discussed our judgements. Of the 48 outcomes that were included across the 12 examples, we were not able to reach a consensus regarding five. The lack of consensus resulted from disagreement about whether there was sparse data for three outcomes and because of insufficient information for two outcomes.

**Table 4:** Results, summary of the judgements made by the 17 evaluators of the quality for each of the outcomes presented in the 12 examples in the pilot study.

Outcome	High	Moderate	Low	Very low	Consensus	Comments
Depression severity	6/17	10/17	-	-	Moderate	
Transient side effects	7/17	10/17	-	-	High	Changed to little uncertainty
Poisoning fatalities	-	4/17	7/17	6/17	Moderate	Upgraded for very strong association
Stroke	15/17	2/17	-	-	High	
Extracranial hemorrage	16/16	-	-	-	High	
All cause mortality	12/17	5/17	-	-	-	Agreed to remove this outcome
Pain at rest	16/17	1/17	-	-	High	
Pain ay motion	15/17	2/17	-	-	Moderate	Uncertainty about directness of outcome measure
Mobility	3/17	14/17	-	-	Moderate	
Quality of life	1/17	11/17	5/17	-	Moderate	
Dropout due to side effects	14/17	3/17	-	-	High	
Serious gi complications	-	3/17	8/17	6/17	-	Need more information before consensus
All cause mortality	2/17	15/17	-	-	Moderate	
Non-fatal stroke	17/17	-	-	-	High	
Non-fatal MI	17/17	-	-	-	High	
Death	13/17	4/17	-	-	High	
Non-fatal MI	11/16	5/16	-	-	High	
All cause death	12/17	5/17	-	-	Moderate	If reporting bias, otherwise high
Major bleeding	15/17	2/17	-	-	High	
Recurrent thromboembolism	6/17	11/17	-	-	High	
Clinical cure	-	13/17	4/17	-	Moderate	
Dropout due to side effects	-	10/17	7/17	-	Moderate	
Relapse	2/17	11/17	4/17	-	Moderate	
Tuberculosis	2/17	10/17	5/17	-	Moderate	
TB death	8/17	8/17	1/17	-	High	
TB meningitis	1/17	4/17	12/17	-	Moderate	Strong association
Serious adverse events	1/12	-	-	11/12	-	No data, outcome removed
Condition unchanged	5/17	19/17	2/17	-	-	No consensus regarding sparse data
Poor outcome- surgeon rated	9/17	8/17	-	-	-	Need bias information before consensus
2^nd ^procedure needed	9/17	8/17	-	-	Moderate	
No success – objective rater	5/17	10/17	2/17	-	-	No consensus regarding sparse data
Risks & side effects	1/15	2/15	2/15	10/15	-	No data, outcome removed
Dental caries – start	-	-	8/17	9/17	Very low	
Dental caries – stop	-	-	5/17	12/17	Very low	
Dental florosis	-	1/17	3/17	13/17	Very low	
Bone fracture	-	1/17	3/17	13/17	Very low	
Cancer mortality	-	1/17	2/17	14/17	Very low	
All injuries	-	1/17	12/17	4/17	Very low	Question changed
Correct use early	8/17	5/17	4/17	-	High	Question changed
Correct use follow up	2/17	8/17	6/17	1/17	High	Question changed
Possession of seat	7/17	6/17	3/17	-	High	Question changed
CHD	11/13	2/13	-	-	High	
Breast cancer	11/13	2/13	-	-	High	
Stroke	11/13	2/13	-	-	High	
Colorectal cancer	11/13	2/13	-	-	High	
Endometrial cancer	11/13	2/13	-	-	High	
Hip fracture	11/13	2/13	-	-	High	

We found that in addition to study design, quality, consistency and directness, other quality criteria also influenced judgements about evidence. These additional criteria were sparse data, strong associations, publication bias, dose response, and situations where all plausible confounders strengthened rather than weakened our confidence in the direction of the effect. Concequently, the consistency with which we considered these additional issues were affected and disagreements regarding the quality of evidence for each outcome were reduced.

### Relative importance of each outcome

Specification of outcomes in the question that each example addressed resulted in some confusion regarding the relative importance of each outcome and the overall quality of evidence across outcomes. We therefore agreed that outcomes should not be included in the questions and that all important outcomes should be considered. There was good agreement about the relative importance of the 48 outcomes that were considered. We reached a consensus about the relative importance of all but two of the outcomes. This was due to uncertainty and true disagreement about the importance of these two outcomes, dental fluorosis and bone fractures, in relation to the question about water fluoridation.

### Overall quality of important outcomes

There was a lack of agreement about the overall quality of evidence across the critical outcomes for each question (Table 5). This poor agreement reflected an accumulation of disagreements about the quality of evidence and importance of the individual outcomes that were considered for each question. In addition, we found that it did not make sense to downgrade the overall quality of evidence because of lower quality evidence for one of several critical outcomes when all of the outcomes showed effect in the same direction. We therefore agreed that the overall quality of evidence should be based on the higher quality evidence, rather than the lowest quality of evidence, when all of the results are in favour of the same option.

**Table 5:** Results, summary of the judgements made by the 17 evaluators of the overall quality in the 12 examples in the pilot study

**Example**	**High**	**Moderate**	**Low**	**Very low**	**Consensus**	**Comments**
1	2/15	4/15	5/15	4/15	Moderate	
2	12/17	5/17	-	-	High	
3	1/17	6/17	5/17	5/17	Need more information before consensus	
4	4/17	13/17	-	-	High	Based on the new rule
5	12/16	4/16	-	-	High	
6	7/17	10/17	-	-	High	Based on new rule
7	-	11/17	6/17	-	Moderate	
8	-	6/17	3/17	8/17	High	Based on new rule
9	2/16	3/16	5/16	6/16	High/Moderate depending if there are fatal flaws	
10	-	1/17	4/17	12/17	Very low	
11	1/17	3/17	8/17	5/17	High	Changed question
12	11/13	2/13	-	-	High	

The kappa statistics for each question are shown in Table 6. The number of outcomes per example range from two to seven and the kappa ranged from 0 to 0.82. In some instances, the agreement among the graders was slightly worse than by chance as indicated by the negative kappa values seen in Table 6. The kappa across the 46 outcomes included in the calculation was 0.395 (SE 0.008). Kappa for agreement beyond chance for the 12 final judgements about the quality of evidence was 0.270 (SE 0.015).

**Table 6:** Results, kappa agreement among the evaluators for each of the 12 examples in the pilot study

**Example**	**No of outcomes**	**P**	**Kappa**	**(SE)**
1	3	0.436	0.149	0.031
2	3	0.769	0.075	0.053
3	6	0.643	0.441	0.024
4	3	0.926	0.823	0.050
5	2	0.608	-0.044	0.065
6	3	0.618	0.163	0.050
7	3	0.520	-0.028	0.044
8	3	0.451	0.146	0.036
9	4	0.441	-0.022	0.037
10	5	0.579	0.005	0.034
11	4	0.377	0.112	0.027
12	7	0.718	-0.083	0.043

### Balance between benefits and harms

The graders assessments about the balance between benefits and harms are shown in Table 7. There is visibly a poor agreement, this can, in part, be explained by the accumulation of all the previous differences in grading of the quality and importance of the evidence. Some of the judges made assumptions or considered information that was not included in the evidence profiles. When we discussed these judgements, we reached a consensus about the balance between benefits and harms for all but three questions. For one question we found we needed more information. For the second judgement we disagreed about the importance of two of the outcomes. For the third judgement we disagreed about the relative values we attached to the benefits and the harms.

**Table 7:** Results, summary of the judgements made by the 17 evaluators about the balance between benefits and harms for each of the 12 examples in the pilot study

Example	Net benefit	Trade offs	Uncertain net benefits	Not net benefits	Consensus
1	10/16	2/16	3/16	1/16	Net benefit
2	11/16	4/16	1/16	-	Net benefit
3	2/17	8/17	7/17	-	Need more information
4	15/16	-	1/16	-	Net benefit
5	13/17	-	4/17	-	Net benefit
6	13/17	2/17	2/17	-	Net benefit
7	4/17	3/17	9/17	1/17	Uncertain net benefits
8	7/16	-	9/16	-	Net benefit
9	2/16	8/16	6/16	-	Uncertain benefit/trade offs
10	2/17	4/17	10/17	1/17	No consensus
11	12/17	-	5/17	-	Net benefit
12	-	2/13	1/13	10/17	No consensus

### Recommendation

The graders individual considerations about the recommendations are shown in Table 8. During the discussion, we reached a consensus on a recommendation for the nine examples where we agreed on the balance between benefits and harms. We found that first agreeing on the balance between the benefits and harms clarified our judgements about recommendations and facilitated a consensus. There was not a one-to-one correspondence between our judgements about trade-offs and our judgements about recommendations, because the latter took into account additional considerations.

**Table 8:** Results, summary of the recommendations made the 17 evaluators for each of the 12 examples in the pilot study

Example	Do it	Probably do it	Toss up	Probably don't do it	Don't do it	Consensus
1	4/16	7/16	3/16	-	2/16	Probably do it
2	6/16	8/16	2/16	-	-	Do it
3	-	6/15	7/15	2/15	-	Need more information
4	13/15	2/15	-	-	-	Do it
5	11/16	5/16	-	-	-	Do it
6	11/17	5/17	1/17	-	-	Do it
7	1/17	7/17	2/17	6/17	1/17	Probably do it
8	2/15	7/15	4/15	2/15	-	Do it
9	1/17	4/17	8/17	4/17	-	Probably don't do it/Tossup
10	-	2/17	6/17	7/17	2/17	No consensus
11	7/17	8/17	2/17	-	-	Do it
12	-	-	-	4/13	9/13	No consensus

### Sensibility and understandability

Eleven raters provided feedback on the sensibility and understandability of the GRADE system for grading evidence and formulating recommendations. Nine of the 11 respondents agreed or strongly agreed that the judgements about the overall quality of evidence were clear and understandable, and that the judgements about the balance between benefits and harms were clear and understandable using the GRADE approach. Everyone agreed or strongly agreed that the judgements about recommendations were clear and understandable. Eight of the judges agreed or strongly agreed that the GRADE approach to judging the overall quality of evidence was better than other grading systems with which they were familiar. Two disagreed and one was not sure. Eight also agreed that the GRADE approach to formulating recommendations was better than approaches with which the raters were familiar. Three raters were not sure about whether the GRADE approach was superior to other approaches of formulating recomendations.

Nine of the 11 respondents agreed or strongly agreed that the GRADE approach was applicable to different types of interventions, and that the approach was clear and simple to apply. Five judges disagreed that the information that is needed is generally available, two were not sure and four agreed. Six of the eleven judges disagreed or strongly disagreed that subjective decisions were generally not needed, four were not sure and one agreed. Ten of the eleven judges agreed or strongly agreed that all the components included in each of the four types of judgements should be included; one judge was not sure. Five of the judges were unsure if there were not important components that were missing from any of the four types of judgements, one disagreed and three agreed or strongly agreed. Eight judges agreed or strongly agreed that the ways in which the components were aggregated for each of the four types of judgements were clear and simple; three were unsure. Seven judges agreed or strongly agreed that the ways in which the included components were aggregated were appropriate for each of the four types of judgements, two were unsure and two disagree. Ten of the eleven judges agreed or strongly agreed that the categories were sufficient to discriminate between different grades for each of the four types of judgements; one disagreed. All the eleven judges agreed or strongly agreed that the GRADE approach successfully discriminated between different quality of evidence, and between different grades of recommendations.

## Discussion

This pilot study of the GRADE approach to grading the quality of evidence and strength of recommendations helped to identify problems with the approach and enabled us to address these. We found that it was possible to resolve most of the disagreements we had when making judgements independently and there was agreement that this approach warrants further development and evaluation.

Many of the disagreements were a direct result of a lack of information. We concluded that there is a need for detailed additional information in evidence profiles, and have modified the evidence profiles accordingly. When we have found an empirical basis or compelling arguments, we have also provided precise definitions. For example, we have agreed on a basis for defining strong and very strong associations. However, in many cases we continue to rely on judgement. We have addressed this by always including the rationale for such judgements in footnotes attached to the evidence profile.

The evidence profiles used in the pilot study were based on systematic reviews. [2-13] Much of the information we found lacking was missing in these original systematic reviews, particularly information about harms and side effects. It was outside of the scope of this study to systematically collect this information. However, systematic reviews of evidence of harms, as well as benefits, are essential for guidelines development panels. If reviews, such as Cochrane reviews, are going to meet the needs of guideline development panels, and others making decisions about health care, it is essential that evidence of adverse effects is systematically included in these.

An important benefit of the approach to grading evidence and recommendations that we used in this study is that it clarifies the source of true disagreements, as well as helping to resolve disagreements through discussing each type of judgement sequentially. Judgements about the relative importance of different outcomes and about trade-offs, as well as about the quality of evidence, are made explicitly, rather than implicitly. This facilitates discussion and clarification of these judgements. It may be helpful to guideline panels and others to use this approach before making decisions and recommendations.

The most common source of disagreement that we encountered was differences in what we consider to be sparse data. We have not reached a consensus on a definition of sparse data, but have acknowledged that we have different thresholds and now recognize this when we make judgements about the quality of evidence [16].

We have as a result of this pilot study been able to make considerable improvements to our system for grading the quality of evidence and strength of recommendations. The evidence profiles used in the pilot study have been modified and now include information that was missing and was found to be an important source of disagreement, as illustrated in Table 9 and Table 10 and the criteria used for grading the quality of evidence for each important outcome have been modified as summarised in Table 11. Guideline generation includes judgement. Individual, residual judgements will impact on the agreement we measured in this study. Thus, lower kappa values are expected. Further refinement of the GRADE system and additional instructions will improve agreement.

**Table 9:** Example of a modified GRADE evidence profile quality assessment. Table 9 and 10 is what Table 1 and 2 became when including the improvements made based on the pilot study experience. Question: Should depressed patients be treated with SSRIs rather than tricyclics? Setting: Primary care Patients: Moderately depressed adult patients Reference: North of England Evidence Based Guideline Development Project. Evidence based clinical practice guideline: the choice of antidepressants for depression in primary care. Newcastle upon Tyne: Centre for Health Services Research, 1997.

Outcome: **Depression severity **(measured with Hamilton Depression Rating Scale after 4 to 12 weeks)									
Studies	Design	Quality	Consistency	Directness	SD	SA	RB	DR	PC

8 trials Citalopram 38 trials Fluoxetine 25 trials Fluvoxamine 2 trials Nefazodone 18 trials Paroxetine 4 trials Sertaline 4 trials Velafaxine	RCTs	No serious limitations	No important inconsistency	Some uncertainty about directness (outcome measure)*	No	No	No	No	No

Outcome: **Transient side effects resulting in discontinuation of treatment**									

8 trials Citalopram 50 trials Fluoxetine 27 trials Fluvoxamine 4 trials Nefazodone 23 trials Paroxetine 6 trials Sertaline 5 trials Velafaxine	RCTs	No serious limitations	No important inconsistency	Direct	No	No	No	No	No

Outcome: **Poisoning fatalities**									

Office for National Statistics (British)	Observational data	Serious limitation**	Only one study	Direct	No	++	No	No	No

*There was uncertainty about the directness of the outcome measure because of the short duration of the trials.

**It is possible that people at lower risk were more likely to have been given SSRI's and it is uncertain if changing antidepressant would have deterred suicide attempts.

SD = Sparse data (Yes or No)

SA = Strong association (No, + = strong, ++ = very strong)

RB = Reporting bias (Yes or No)

DR = Dose response (Yes or No)

PC = All plausible confounders would have reduced the effect (Yes or No)

CI = confidence interval

WMD = weighted mean difference

RRR = relative risk reduction

**Table 10:** Example of a modified GRADE evidence profile summary of findings. Table 9 and 10 is what Table 1 and 2 became when including the improvements made based on the pilot study experience

**Outcome**	**SSRI**	Tricyclics	**Effect**		**Quality**	**Importance**
					
			**Relative (95% CI)**	**Absolute**		
Depression severity	5044 patients	4510 patients	WMD 0.034 (-0.007 to 0.075)	No difference	Moderate	Critical
Transient side effects	1948/7032 (28%)	2072/6334 (33%)	RRR 13% (5% to 20%)	5 per 100	High	Critical
Poisoning fatalities***	1/100,000 per year of treatment	58/100,000 per year of treatment	RRR 98% (97% to 99%)	6 per 10,000	Moderate	Critical

***There is uncertainty about the baseline risk for poisoning fatalities.

**Table 11:** Modified GRADE quality assessment criteria

**Quality of evidence**	**Study design**	Lower if *	Higher if *
High	Randomised trial	**Study quality**: -1-Serious limitations -2-Very serious limitations -1-Important **inconsistency** **Directness**: -1-Some uncertainty -2-Major uncertainty -1-**Sparse data** -1-High probability of **Reporting bias**	**Strong association**: +1-Strong, no plausible confounders, consistent and direct evidence** +2-Very strong, no major threats to validity and direct evidence*** +1-Evidence of a **Dose response **gradient +1-All **plausible confounders **would have reduced the effect
		
Moderate	Quasi-randomised trial		
		
Low	Observational study		
		
Very low	Any other evidence		

* 1 = move up or down one grade (for example from high to moderate)

2 = move up or down two grades (for example from high to low)

The highest possible score is High (4) and the lowest possible score is Very low (1). Thus, for example, randomised trials with a strong association would not move up a grade.

** A relative risk of >2 (< 0.5), based on consistent evidence from two or more observational studies, with no plausible confounders

*** A relative risk of > 5 (< 0.2) based on direct evidence with no major threats to validity

Judgements about confidence in evidence and recommendations are complex. The GRADE system represents our current thinking about how to reduce errors and improve communication of these complex judgements. Ongoing developments include:

• Exploring the extent to which the same system should be applied to public health and health policy decisions as well as clinical decisions

• Developing guidance for when and how costs (resource utilisation) should be considered

• Developing guidance for judgements regarding sparse data

• Adapting the approach to accommodate recommendations about diagnostic tests when these are based on evidence of test accuracy

• Incorporating considerations about equity

• Preparing tools to support the application of the GRADE system

Plans for further development include studies of the reliability and sensibility of this approach and a study comparing alternative ways of presenting these judgements [17]. We invite other organisations responsible for systematic reviews of the effects of healthcare or practice guidelines to work with us to further develop and evaluate the system described here.

## Conclusion

Based on the results of this pilot study we have been able to considerably improve our system for grading the quality of evidence and strength of recommendation [16].

## Competing interests

DA has competing interests with the US Preventive Services Task Force (USPSTF), PAB has a competing interest with the US Task Force on Community Preventive Services (USTFCPS), ME has and JM had competing interests with the National Institute for Clinical Excellence (NICE), GHG, RJ and HS have competing interests with the American College of Chest Physicians (ACCP), RTH has competing interests with the Scottish Intercollegiate Guidelines Network (SIGN), SH and DO'C have competing interests with the Australian National Health and Medical Research Council (ANHMRC), BP has competing interests with the Oxford Centre for Evidence-Based Medicine (OCEBM). Most of the other members of the GRADE Working Group have experience with the use of one or more systems for grading evidence and recommendations.

## Authors' contributions

DA, PAB, ME, SF, GHG, RTH, SH, RJ, AL, NM, JM, DO'C, ADO, BP, HS, TTTE, GEV & JWJ Jr as members of the GRADE Working Group have contributed to the preparation of this manuscript and the development of the ideas contained herein, participated in the pilot study, and read and commented on drafts of this article. All authors except GEV judged the quality of the evidence and strength of recommendation based on information presented in the evidence profiles. GEV prepared the first draft of this article, had primary responsibility for preparing the evidence profiles used in the study, and coordinated the study.

## Pre-publication history

The pre-publication history for this paper can be accessed here:



## Supplementary Material

Additional File 1 Instructions and form for judgements used in the pilot study Click here for file
