# Supplementary material for: Systems for grading the quality of evidence and the strength of recommendations II: Pilot study of a new system
Source: BMC Health Serv Res. 2005 Mar 23;5:25. doi: 10.1186/1472-6963-5-25 (PMC1084246; doi:10.1186/1472-6963-5-25)
Supplement: Additional File 1 — Instructions and form for judgements used in the pilot study [file 1472-6963-5-25-S1.doc]

**Additional file 1. Instructions and form for judgements used in the pilot study**

# Please read the information and instructions below carefully before starting

# Instructions

The evidence profile has been prepared based on information from a systematic review or a practice guideline. It may not contain all the information or studies that you may consider relevant for the question asked.

Do not second-guess what is in the table. Use only the information presented in the table as the basis for your judgements.

Please note any disagreements and comments you may have about the table.

1. Quality of evidence across studies for each outcome

When grading the quality of evidence for each outcome (across studies), please refer to and use the following guidelines.

| **Quality of**  **evidence** | **Study**  **design** | **Lower if *** | **Higher if *** |
| --- | --- | --- | --- |
| **High** | **RCT** | Study Quality**:**  **1-Serious flaws**  **2-Very serious flaws**  Consistency**:**  **1-Important inconsistency**  Directness**:**  **1-Some uncertainty**  **2-Major uncertainty**  Reporting bias  Sparse data | Association**:**  **2-Extremely strong**  **2-Very strong (no plausible confounders, consistent and direct)**  **1-Strong (no plausible confounders, consistent and direct)** |
| **Moderate** | **Quasi-RCT** |
| **Low** | **Observational study** |
| **Very low** | **Any other evidence** |

*** 1 = move up or down one grade (for example from high to moderate)**

**2 = move up or down two grades (for example from high to low)**

2. Relative importance of outcomes

Please indicate for each of the outcomes presented, the relative importance of this outcome for making a decision about the question posed at the top of the evidence table.

Outcomes with a mean of 7 or above will be considered critical outcomes

Outcomes with a mean between 4 and 6 will be considered important, but not critical to the decision. These should be used in judgements about tradeoffs and recommendations, but not in judgements about the overall quality of evidence across the critical outcomes.

Outcomes with a mean of 3 or below will be removed from the evidence profile and should NOT be considered further in the judgements about the overall quality of evidence, tradeoffs or recommendations.

3. Overall quality of evidence across the critical outcomes

The overall quality of the evidence across the critical outcomes, should be based on the lowest quality of evidence for the outcomes that are critical to making a decision.

4. Balance of the benefits and harms

Based on the critical and other important outcomes, what is the balance between benefits and harms? Your judgements about the balance between benefits and harms and the recommendations should NOT take into account costs.

5. Recommendation

This should be based on the

- trade-offs
- quality of evidence (for critical outcomes)
- translation of evidence into practice in the specific situation (if specified)
- uncertainty about baseline risk

Rules of thumb for making judgements about recommendations are

90 - 100% of people likely to do it = do it

60 - 90% of people likely to do it = probably do it

40 - 60% of people likely to do it = maybe do it

10 - 40% of people likely to do it = probably don’t do it

0 - 10% of people likely to do it = don’t do it

6. Comments and suggestions

Please note comments and suggestions regarding this particular evidence profile or generally about how the evidence profile can be improved.

Please specify any special circumstances that should have been specified, but were not.

# Data collection form for GRADE pilot

**Antidepressants**

Question: Should depressed patients in primary care be treated with SSRIs rather than tricyclics?

Name: _______________________________________________

Date: _______________________________________________

1. For each of the following outcomes indicate the quality of evidence across studies:

Depression severity _________________________________________________________

🞏 High 🞏 Moderate 🞏 Low 🞏 Very low

COMMENTS:

Transient side effects _________________________________________________________

🞏 High 🞏 Moderate 🞏 Low 🞏 Very low

COMMENTS:

Poisoning fatalities _________________________________________________________

🞏 High 🞏 Moderate 🞏 Low 🞏 Very low

COMMENTS:

1. Please indicate the relative importance of each outcome (by circling a number between 1 and 9).
   - Depression severity ___________________________________________________

Not critical to making 1 2 3 4 5 6 7 8 9 Critical to making

a decision a decision

- - Transient side effects

___________________________________________________

Not critical to making 1 2 3 4 5 6 7 8 9 Critical to making

a decision a decision

- - Poisoning fatalities ___________________________________________________

Not critical to making 1 2 3 4 5 6 7 8 9 Critical to making

a decision a decision

1. What is the overall quality of evidence across the critical outcomes (those with a relative importance of 7-9)?

🞏 High 🞏 Moderate 🞏 Low 🞏 Very low

COMMENTS:

1. What is the balance between the above benefits (desirable outcomes) and harms (undesirable outcomes)?

🞏 Net benefits

🞏 Trade-offs

🞏 Uncertain net benefits

🞏 Not net benefits

COMMENTS:

1. Which of the following recommendations would be appropriate for this intervention?

🞏 Do it

🞏 Probably do it

🞏 “Toss-up”

🞏 Probably don’t do it

🞏 Don’t do it

COMMENTS:

1. To what extent is this an adequate representation of the main results of the review/guideline? (circle a number between 1 and 9 below)

The quality assessment is 1 2 3 4 5 6 7 8 9 The quality assessment

not adequate is adequate

The summary of findings is 1 2 3 4 5 6 7 8 9 The summary of findings

not adequate is adequate

COMMENTS (please specify inadequacies)
